# Impact of PNPase on the transcriptome of *Rhodobacter sphaeroides* and its cooperation with RNase III and RNase E

**DOI:** 10.1186/s12864-021-07409-4

**Published:** 2021-02-06

**Authors:** Daniel-Timon Spanka, Carina Maria Reuscher, Gabriele Klug

**Affiliations:** Institute of Microbiology and Molecular Biology, Justus Liebig University Giessen, IFZ, Giessen, Germany

**Keywords:** Alphaproteobacteria, *Rhodobacter sphaeroides*, PNPase, Exoribonuclease, Transcriptomics, RNA 3′ end identification, RNase E, XPEAP

## Abstract

**Background:**

The polynucleotide phosphorylase (PNPase) is conserved among both Gram-positive and Gram-negative bacteria. As a core part of the *Escherichia coli* degradosome, PNPase is involved in maintaining proper RNA levels within the bacterial cell. It plays a major role in RNA homeostasis and decay by acting as a 3′-to-5′ exoribonuclease. Furthermore, PNPase can catalyze the reverse reaction by elongating RNA molecules in 5′-to-3′ end direction which has a destabilizing effect on the prolonged RNA molecule. RNA degradation is often initiated by an endonucleolytic cleavage, followed by exoribonucleolytic decay from the new 3′ end.

**Results:**

The PNPase mutant from the facultative phototrophic *Rhodobacter sphaeroides* exhibits several phenotypical characteristics, including diminished adaption to low temperature, reduced resistance to organic peroxide induced stress and altered growth behavior. The transcriptome composition differs in the *pnp* mutant strain, resulting in a decreased abundance of most tRNAs and rRNAs. In addition, PNPase has a major influence on the half-lives of several regulatory sRNAs and can have both a stabilizing or a destabilizing effect. Moreover, we globally identified and compared differential RNA 3′ ends in RNA NGS sequencing data obtained from PNPase, RNase E and RNase III mutants for the first time in a Gram-negative organism. The genome wide RNA 3′ end analysis revealed that 885 3′ ends are degraded by PNPase. A fair percentage of these RNA 3′ ends was also identified at the same genomic position in RNase E or RNase III mutant strains.

**Conclusion:**

The PNPase has a major influence on RNA processing and maturation and thus modulates the transcriptome of *R. sphaeroides*. This includes sRNAs, emphasizing the role of PNPase in cellular homeostasis and its importance in regulatory networks. The global 3′ end analysis indicates a sequential RNA processing: 5.9% of all RNase E-dependent and 9.7% of all RNase III-dependent RNA 3′ ends are subsequently degraded by PNPase. Moreover, we provide a modular pipeline which greatly facilitates the identification of RNA 5′/3′ ends. It is publicly available on GitHub and is distributed under ICS license.

**Supplementary Information:**

The online version contains supplementary material available at 10.1186/s12864-021-07409-4.

## Background

Prokaryotes populate nearly every imaginable habitat. In contrast to higher multicellular eukaryotes, they are directly exposed to all types of environmental stress. Since escaping is not an option, prokaryotes need mechanisms to quickly adapt to their changing surrounding. This can be achieved by modifying the transcriptome and/or the proteome. One essential mechanism in bacterial adaptation is to exchange the sigma factor, a subunit of the RNA polymerase. Alternative sigma factors target different DNA sequences and thus activate the expression of a specific set of genes. This activates transcription of genes needed for the cell to deal with the present growth condition [1, 2].

Besides and in addition to the transcriptional initiation, posttranscriptional regulation plays a major role in bacterial adaptation [3]. During the past decades, more and more bacterial non-coding RNAs were discovered and found to be involved in various posttranscriptional regulatory networks (reviewed in [4]).

Current studies documented, that the prokaryotic transcriptome is heavily influenced by processing and maturation reactions mediated by the endoribonuclease E [5–7]. Endonucleolytic RNA cleavage by RNase E is mostly followed by further degradation. 3′-to-5′ exonucleases can attack the new 3′ end and RNase E can bind to the monophosphorylated new 5′ end and promote further endonucleolytic degradation in 5′-to-3′ direction. Secondary structures can protect against 3′-to-5′ degradation [8] and can also impede RNase E mediated 5′-to-3′ processing [9]. A key player during RNA turnover is a multicomponent degradation complex called the degradosome. In *Escherichia coli*, this complex is composed of RNase E, which serves as catalytic and scaffold protein, a DEAD box RNA helicase (RhlB), an exoribonuclease (polynucleotide phosphorylase, PNPase) and an enolase (reviewed in [10]). In contrast to that, studies of the α-proteobacterium *Rhodobacter capsulatus* suggest, that PNPase is most likely not part of its degradosome [11] that in addition to RNase E includes 2 dead-box helicases and the transcriptional termination factor Rho. Moreover, the composition of the *R. capsulatus* degradosome changes in response to altering environmental conditions [12].

The PNPase is a trimer comprising three Pnp monomers that form a ring-like structure. In *E. coli*, each monomer consists of two RNase PH-like domains and a KH and S1 domain [13, 14]. A deletion of *pnp* is possible in *E. coli*, whereas a double knockout of PNPase and RNase II is not viable [15]. The *R. sphaeroides* genome does not harbour an RNase II gene and it is not possible to delete the *pnp* gene. The same effect was also observed in at least one other organism, *Pseudomonas aeruginosa* [16]. Removal of the RNA-binding KH/S1 domains of PNPase leads to an eightfold reduced binding affinity to RNA in *E. coli* [14]. Further, the trimer formation is less stable, which leads to a wider central channel [14]. PNPase not only serves as an important 3′-to-5′ exoribonuclease involved in mRNA degradation but also in tRNA processing and degradation [15, 17]. Besides that, PNPase can also prolong RNA molecules in 5′-to-3′ direction using nucleotide diphosphates present in the cytoplasm. This tail allows recruitment of single-strand dependent exoribonucleases thus reducing the RNA half-life [18]. Since PNPase is an enzyme with such a widespread influence on the cellular RNAs, the *pnp* expression has to be tightly regulated. Similar to *rne* mRNA levels, *pnp* mRNA levels are balanced in an autoregulatory manner. The endoribonuclease RNase III first cleaves a stem-loop located in the *pnp* leader sequence. The newly generated 3′ end in this RNA duplex is then targeted and degraded by PNPase. Ultimately this leads to reduced *pnp* mRNA stability [19, 20]. Besides PNPase, several other exoribonucleases are likely involved in RNA processing, maturation and degradation in the α-proteobacterium *R. sphaeroides*. These are the RNase R, RNase D and RNase PH which catalyze mainly tRNA and rRNA processing reactions and all act in 3′-to-5′ direction [21–23]. In addition, RNase J1 is responsible for the maturation of the 23S rRNA and very few other transcripts [24, 25]. In contrast to the other RNases, it processes RNA molecules in 5′-to-3′ end direction [26]. The endoribonucleases RNase E, III and G (homolog of RNase E) are mainly responsible for RNA maturation and turnover [7, 27, 28].

**Fig. 1:**
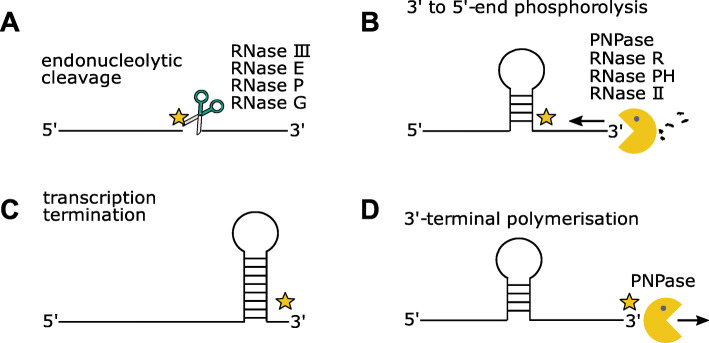
Generation of RNA 3′ ends in bacteria and action of PNPase. **a** RNA 3′-OH ends (highlighted by yellow stars) can be generated via endonucleolytic cleavage by RNase III/E/P/G, **b** by 3′-to-5′ degradation mediated by PNPase and RNase R/PH/II, or **c** by transcriptional termination. **d** PNPase can also produce new 3′-OH ends by a 3′-terminal oligonucleotide polymerase reaction

In order to understand bacterial adaptation, it is important to elucidate the complex interplay between different RNases and how they sequentially process RNA molecules. A common way for degradation of mRNA and maturation of RNA precursors requires two steps: First, the endoribonucleases III, E or P catalyse the endonucleolytic cleavage of the RNA molecule. Second, the enzymes PNPase, RNase R, RNase PH or RNase II can further degrade the RNA fragments from 3′-to-5′-direction (reviewed in [29, 30]). In both steps, new RNA 3′ ends are generated (Fig. 1a+b). Recent studies in the Gram-positive human pathogen *Streptococcus pyogenes* illustrate how initial processing by endoribonuclease Y is followed by further maturation reactions catalyzed by the exoribonucleases PNPase and RNase R [31]. The other principal mechanisms for RNA 3′ end generation are transcription termination by RNA polymerase and the 3′-terminal elongation mediated by PNPase (Fig. 1c + d).

In this study, we report that in the *Rhodobacter sphaeroides pnp* mutant strain several physiological characteristics are affected by the deletion of the KH and S1 domains, including growth behavior and pigmentation. In a global approach, we further used RNA-Seq data and identified all RNA 3′ ends that are PNPase-, RNase III- or RNase E-dependent. Intersection analysis sheds light on important processing events by the analyzed RNases that shape the transcriptome in a cooperative manner. Finally, we could demonstrate, that homeostasis of the regulatory sRNAs CcsR1–4 rely on initial RNase E cleavage followed by PNPase degradation.

## Methods

### Bacterial strains and growth conditions

The strains used in this study are listed in Table S1 [32]. Microaerobic *Rhodobacter sphaeroides* cultures (dissolved oxygen concentration of 25–30 μM) were cultivated in 50 ml Erlenmeyer flasks filled with 40 ml malate minimal medium at 32 °C under continuous shaking at 140 rpm in the dark [33]. To perform phototrophic cultivation, Metplat bottles were completely filled and sealed. Afterwards the cultures were constantly exposed to white light with an intensity of 40 W/m^2^ at 32 °C.

### Construction of *pnp* KH and S1 deletion strain

**Fig. 2:**
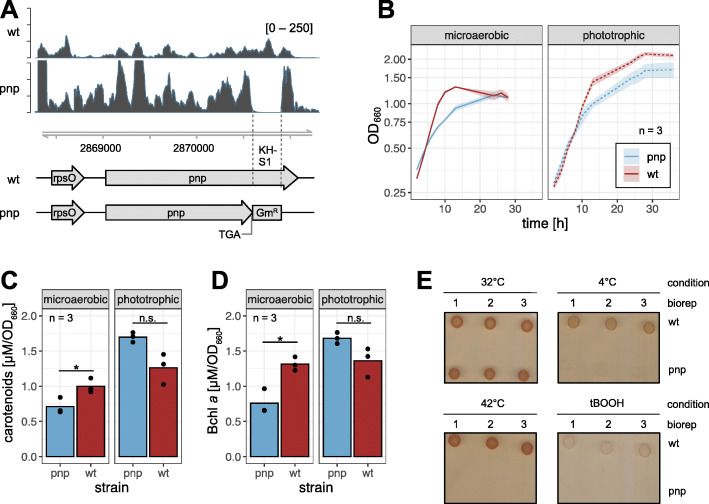
The *pnp* mutant and the wild type strain differ in growth behaviour, pigmentation as well as in growth under different temperatures and under organic peroxide stress. **a** Schematic overview of the *pnp* operon. In the *pnp* mutant, the KH-S1 domains were deleted and substituted with a gentamicin resistance gene. A stop codon was inserted at the end of the remaining *pnp* coding region. Upper panels show the RNA read coverage in the wild type and *pnp* mutant strain. **b** The *pnp* mutant grows slower than the wild type under microaerobic cultivation and does not reach the wild type optical density during stationary phase when cultivated under phototrophic conditions. Red: wild type; blue: *pnp* mutant; *n* = 3. **c, d** Exponentially growing *pnp* mutant cultures exhibit reduced carotenoid and bacteriochlorophyll *a* (Bchl *a*) concentrations under microaerobic conditions in comparison to the wild type strain. Phototrophically cultivated, the pigment concentrations are increased in the mutant. The *p*-values were calculated using two-sided Student’s *t*-test (*: *p* < 0.05; n.s.: not significant). **e** On solid malate minimal agar, the growth of the *pnp* mutant strain is strongly impaired when the plates are incubated at 4 °C or 42 °C. The organic peroxide tBOOH (300 μM final concentration) diminishes growth of the wild type but prevents growth of the *pnp* mutant strain. Biological triplicates are shown for each growth condition

The deletion of the *pnp* C-terminal KH and S1 domains in *Rhodobacter sphaeroides* 2.4.1 was carried out by homologous recombination. Since *pnp* is essential in *R. sphaeroides*, only the RNA binding domains KH/S1 were replaced by a gentamicin resistance gene on the chromosome. The up and down fragments were generated using the primer pairs pnpFragAfw/pnpFragArev (5′-gaaTTCAAGAAGCTGGAAAGCTCGAT, 5′-ggatcctcAGGTTTCCACGATCTCGCGG, 870 bp) and pnpFragBfw/pnpFragBrev (5′-ggaTCCGTCTCGGCATGAAGATG, 5′-aagcTTCTCGTCCGAAGACGTGCTG, 631 bp), introducing an in-frame TGA stop codon within the reverse primer of the up fragment (see underlined bases in primer pnpFragArev). The stop codon is located at position 1755 in the *pnp* gene and leads to translation termination directly upstream of the deleted KH/S1 region (Fig. 2a). Both fragments were cloned in pPHU281 using EcoRI/BamHI and BamHI/HindIII cleavage sites. The gentamicin resistance gene was taken from pPHU45Ω and inserted between the up and down fragment on the plasmid with BamHI. The final construct was transformed to *E. coli* strain S17–1 and subsequently transferred to *Rhodobacter sphaeroides* 2.4.1 by diparental conjugation. The conjugants were selected on malate minimal agar containing 10 μg/ml gentamicin.

### Measurement of bacteriochlorophyll and carotenoids

The determination of bacteriochlorophyll and carotenoid concentrations was performed as described in [34]. The calculations rely on the extinction coefficients (76 mM^− 1^·cm^− 1^ for bacteriochlorophyll *a*, 128 mM^− 1^·cm^− 1^ for carotenoids) published in [35].

### Spot assay

A volume of 5 μl taken from a liquid culture during the exponential growth phase was placed on malate minimal agar plates. The plates were first incubated at 4 °C or 42 °C for 1 day and then shifted to 32 °C and cultivated for three more days. To test resistance to organic peroxides, *tert*-butyl hydroperoxide (tBOOH) was added to the agar (300 μM final concentration). That plate as well as the control without any tBOOH were subsequently incubated at 32 °C for 3 days.

### Determination of RNA half-life

*Rhodobacter* cultures were cultivated under microaerobic conditions. During the exponential phase, the sample t_0_ was harvested. Immediately after that the transcription inhibitor rifampicin was added to a final concentration of 0.2 mg/ml. The following samples were taken at the indicated time points. All cells were harvested on ice and total RNA was isolated and blotted as described below.

### Northern blot analysis

The hot phenol method was used to isolate total RNA [36]. The procedure was followed by a DNase treatment (Invitrogen #AM1907) according to the manufacturer’s protocol to digest remaining DNA fragments. The electrophoretic separation in a gel and subsequent Northern blot analysis was performed as described earlier [37]. The oligonucleotide end-labelling was performed using T4 polynucleotide Kinase (T4-PNK, Thermo Scientific) according to the manufacturer’s instructions. Radioactive [γ^32^P]-ATP was obtained from Hartmann Analytic (SRP-301), the oligonucleotides used for labeling are listed in Table S2 in Additional file 1. After overnight incubation with labeled oligonucleotides, the membrane was washed in 5x SSC buffer and exposed to a screen for 1 day. The QuantityOne 1-D Analysis Software (BioRad, version 4.6.6) was used to quantify the signals. All signal intensities were normalized to the corresponding 5S rRNA signal.

### Library preparation

Three single colonies were used to inoculate three independent pre-cultures. Every culture was then used to inoculate three main cultures (nine in total). During the exponential growth phase, all three replicates belonging to one biological pre-culture were harvested and pooled. Total RNA was extracted followed by DNase treatment.

RNA quality was checked using a 2100 Bioanalyzer with the RNA 6000 Nano kit (Agilent Technologies). Five hundred nanograms of high quality total RNA were used for the preparation of a cDNA library with the NEBNext Multiplex Small RNA Library Prep kit for Illumina (NEB) in accordance with the manufacturers’ instructions with modifications: RNA samples were fragmented with Mg^2+^ at 94 °C for 3 min 15 s using the NEBNext Magnesium RNA Fragmentation Module (NEB) followed by RNA purification with the Zymo Oligo Clean & Concentrator kit. Fragmented RNA was dephosphorylated at the 3′ end, phosphorylated at the 5′ end and decapped using 10 U T4-PNK +/− 40 nmol ATP and 5 U RppH, respectively (NEB). After each enzymatic treatment RNA was purified with the Zymo Oligo Clean & Concentrator kit. The RNA fragments were ligated for cDNA synthesis to 3′ single-read (SR) adapter and 5′ SR adapter diluted 1:2 with nuclease-free water before use. PCR amplification to add Illumina adaptors and indices to the cDNA was performed for 14 cycles. Barcoded DNA Libraries were purified using magnetic MagSi-NGS^PREP^ Plus beads (AMSBIO) at a 1.5 ratio of beads to sample volume. Libraries were quantified with the Qubit 3.0 Fluorometer (ThermoFisher) and the library quality and size distribution was checked using a 2100 Bioanalyzer with the DNA-1000 kit (Agilent). Sequencing of pooled libraries, spiked with 10% PhiX control library, was performed in single-end mode on the NextSeq 500 platform (Illumina) with the High Output Kit v2.5 (75 Cycles). Demultiplexed FASTQ files were generated with bcl2fastq2 (Illumina). The sequencing data are available at NCBI Gene Expression Omnibus (http://www.ncbi.nlm.nih.gov/geo) under the accession number GSE156818.

### Bioinformatical analysis

The 3′ end analysis was performed with XPEAP, a pipeline programmed for this study. First, the adapter sequences were removed and all raw reads trimmed for quality with Trim Galore (version 0.6.3). All filtered reads were mapped to the *Rhodobacter sphaeroides* 2.4.1 genome (assembly GCF_000012905.2) using READemption (version 0.4.3 [38];) with the mapper segemehl (version 0.2.0 [39];). The DESeq2 package (version 1.26.0 [40];) was used for the normalization of read counts and the full transcriptome analysis. The results were validated with the R package baySeq (version 2.20.0 [41];) with the gene quantification table obtained from READemption. Coverage generation for both full coverage and 3′ end coverage was done with READemption. The 3′ end coverage files were converted to BED file format with Bedops (version 2.4.37) and filtered. All bases without a minimal read coverage of 10 were rejected. Further, all positions with a signal ratio lower than 5% comparing the 3′ end and the full read coverage were excluded. The nucleotide-wise fold changes were calculated with DESeq2 and all nucleotide positions kept which passed the log_2_-fold change cutoff ≤ − 1 or ≥ + 1 and exhibited an adjusted *p*-value (Benjamini-Hochberg algorithm) lower than 0.05. All positions within a maximal distance of three nucleotides were merged to one 3′ end with BEDtools’ subcommand *merge* (version 2.25.0 [42];), the mean log_2_-fold change was computed for every differential 3′ end. BEDtools *intersect* was used to identify genes with overlapping differential 3′ ends and all ends without any overlapping feature were assigned to untranslated regions.

The intersection of the differential 3′ ends between different RNase mutant strains was analyzed with BEDtools *window* using a window size of 1 nt while only matches on the same strand were considered for further analysis. Fisher’s exact test was calculated for all intersection files using BEDtools’ subcommand *fisher*.

XPEAP is published under ISC license and can be accessed via Zenodo/GitHub (DOI: 10.5281/zenodo.8475, https://github.com/datisp/XPEAP). The raw reads and analyzed data from all experiments are deposited on NCBI Gene Expression Omnibus: PNPase and RNase III mutant strains (NCBI GEO accession number: GSE156818) and thermosensitive RNase E mutant strain (NCBI GEO accession number: GSE71844, published in [7]).

For the 3′ elongation analysis, reads that could not be mapped in end-to-end mode with segemehl were mapped with bowtie2 (version 2.2.6) in local mode with option *–very-sensitive-local* and flags *-f -p 24 –no-hd*. Reads with less than 10 nt matching at the 5′ end were rejected. The sequences following the matching regions were extracted with awk (version 4.1.3).

## Results and discussion

### Physiological consequences of altered PNPase activity

To analyze the functionality of PNPase in vivo, we designed and cloned a *pnp* mutant strain of *Rhodobacter sphaeroides* 2.4.1. The KH-S1 RNA binding domains were removed and a stop codon was introduced at the end of the remaining coding sequence of *pnp* resulting in a truncated enzyme lacking those domains. The knockout was confirmed via selection on agar containing gentamicin and subsequent RNA sequencing analysis (Fig. 2a). Growth behavior of this strain differed from that of the wild type (Fig. 2b). When cultivated under microaerobic conditions, the growth rate was reduced, but both wild type and mutant finally reached the identical OD_660_. Under phototrophic conditions the mutant leaves exponential phase earlier than the wild type reaching a lower final OD_660_. A previous study revealed that reduced RNase E activity strongly impeded phototrophic growth of *R. sphaeroides*, while it had no effect on chemotrophic growth [7]. Moreover, the *pnp* mutant and the parental wild type strain vary in pigment composition (Fig. 2c+d). These differences are strongly dependent on the cultivation conditions: A significantly lower concentration of carotenoids and bacteriochlorophyll *a* was observed in the *pnp* mutant under microaerobic conditions (*p*-values < 0.05), while the *pnp* mutant exhibited repeatedly higher pigment concentrations under phototrophic conditions. However, this difference was statistically not significant.

In *E. coli, Yersinia enterocolitica* and *Photorhabdus sp.* PNPase plays an important role in the cold shock response due to selective degradation of mRNAs for cold shock proteins at the end of the acclimation phase to low temperature [43–46]. Based on this observation, we decided to test the *R. sphaeroides* strains for their ability to adapt to low and high temperatures. Wild type and *pnp* mutant cells were incubated at 4 °C or 42 °C on agar plates for 1 day and then shifted to an optimal temperature of 32 °C. In both cases growth of the *pnp* mutant was strongly impeded, while the wild type was able to grow at 42 °C and 4 °C (Fig. 2e). Also, in contrast to the wild type, the *pnp* mutant was not able to grow on malate minimal agar containing 300 μM tBOOH, while the wild type showed weak growth. Tertiary butyl-alcohol is representing organic peroxides that are produced e. g. during photo-oxidative stress.

Our results show that PNPase of *R. sphaeroides* is involved in cold adaptation as other bacterial PNPases and also is strongly impeded in its adaptation to heat. Whether the same molecular mechanisms are responsible for the phenotype as in other bacteria remains to be elucidated. This study for the first time analyses the function of PNPase in a phototrophic bacterium. The effect of PNPase on the bacteriochlorophyll levels and on carotenoid levels depends on growth conditions. Many genes are involved in the formation of photosynthetic complexes and it is not possible to correlate these phenotypic changes to specific changes of the transcriptome. We observed before that a temperature-sensitive variant of RNase E had little effect on growth under microaerobic conditions but strongly impeded phototrophic growth [7]. For the PNPase mutant we observed slower growth under both conditions, phototrophic growth was less affected, in contrast to the *rne* mutant.

### PNPase modulates the transcriptome of *R. sphaeroides*

PNPase is an enzyme involved in many RNA processing reactions, and a global influence on the transcriptome can be expected as also shown for the Gram-positive *S. pyogenes* [31]. For the transcriptome analysis, three pre-cultures of the wild type and the *pnp* mutant strain of *R. sphaeroides* were inoculated with cells from three different single colonies. With each of these pre-cultures, three main cultures were inoculated (nine in total), grown under microaerobic conditions and later harvested during the exponential growth phase. All cultures initially derived from one colony in the first step were pooled. Total RNA was isolated and the DNA-free RNA was sequenced on an Illumina NextSeq 500 platform. The overall reproducibility within the replicates was fair, only one replicate obtained from the wild type strain showed some deviation to the other samples of the group (Supplementary Fig. S1, Additional file 1). In total 98% of the entire variance can be explained by the first two principal components.

**Fig. 3:**
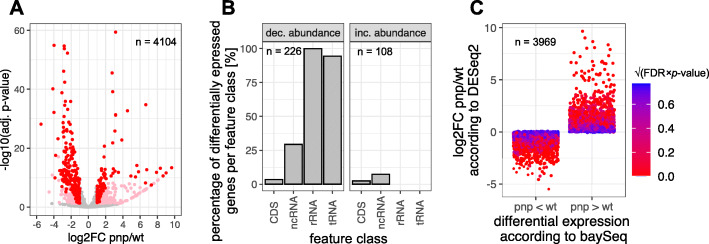
The *Rhodobacter sphaeroides* transcriptome composition is strongly influenced in the PNPase mutant. **a** Volcano plot of the observed log_2_-fold changes based on RNA-Seq data analyzed with DESeq2. Genes with significant change in abundance are colored red (adjusted *p*-value ≤0.05, log_2_fold change ≤ − 1 or ≥ + 1, basemean ≥50) and pink (adjusted *p*-value ≤0.05, log_2_-fold change < − 1 or > + 1, basemean < 50). Grey dots: adjusted *p*-value > 0.05 or − 1 ≤ log_2_-fold change ≥ + 1. Altogether the transcripts of 334 genes were observed to differ in a statistically significant manner and exhibited a basemean above the threshold. **b** Feature-wise distribution of these significant genes, classified in decreased and increased abundance (*pnp* mutant/wild type). Most tRNAs and all rRNAs showed a reduced abundance in the mutant strain. x-axis: feature class; y-axis: percentage of differentially expressed genes per feature class [%] **c**) Comparison of data computed with DESeq2 and baySeq, which show a very good match. Almost all transcripts that are lower abundant in the *pnp* mutant according to DESeq2 (log_2_-fold change (*pnp*/wt) < 0) are also classified to be lower abundant by baySeq (*pnp* < wt) and vice versa. Since baySeq does not provide *p*-values, the color coding represents the square root of the product of the false discovery rate (FDR, obtained from baySeq) and the adjusted *p*-value (obtained from DESeq2). Every dot represents one gene

Figure 3 shows the result of the DESeq2 analysis (version 1.26.0 [40];) and illustrates the log_2_-fold changes of the normalized read numbers in the *pnp* mutant versus the wild type strain (see Supplementary Table S3, Additional file 2). All transcripts with a log_2_-fold change ≤ − 1 or ≥ + 1 and an adjusted *p*-value ≤0.05 (Benjamini Hochberg algorithm) were considered to have a significant differential abundance within the two strains (coloured dots). We then decided to only keep those differentially expressed genes which have a basemean ≥50 (red dots) in order to further decrease the number of false positive hits. In total 334 transcripts met these strict criteria, 226 of them showed lower abundance in the *pnp* mutant strain and 108 showed higher abundance in the *pnp* mutant strain compared to the wild type. The most prominent differences were observed in the feature classes tRNA and rRNA: 94% of all tRNAs (51 out of 54) and 100% of all rRNAs (9 out of 9) showed a lower abundance in the *pnp* mutant strain (Fig. 3b). Altogether 37% of all non-coding RNAs, here merged of sRNAs and ncRNAs (including 6S, SRP RNA and tmRNA), were observed to have a differential abundance. Within the groups of RNAs with increased or decreased abundance, no distinct orthologous group of encoded proteins (COG) could be found to be prominent (Supplementary Fig. S2A + B, Additional file 1).

The transcriptome is directly affected by the action of RNases. Moreover, the RNA entity is modulated through secondary effects by the PNPase-mediated processing of sRNAs and mRNAs that code for regulatory elements, for example transcription factors. Thus, our transcriptome analysis reflects both direct and indirect PNPase dependent regulations and does not allow a distinction. In either case, our data emphasize the effect which PNPase has especially on stable RNAs (rRNA, tRNA). A similar effect was also observed in *E. coli*, although both rRNAs and tRNAs were more abundant in the *pnp* mutant despite a conducted rRNA depletion prior to RNA sequencing [47]. Further, Płociński et al. [48] demonstrated, that PNPase is involved in processing of ribosomal RNA and tmRNA in *Mycobacterium smegmatis* and *M. tuberculosis*.

We further validated these predictions using a different algorithm. An empirical Bayes approach integrated in the baySeq package (version 2.20.0 [41];) was used to identify differential expression (Supplementary Table S4, Additional file 2). The results of the two methods perfectly agree, since virtually all genes could be properly assigned. Every transcript (blue dot) with a log_2_-fold change ≤0 (*pnp* mutant*/*wild type) according to the DESeq2 analysis was also observed to be lower abundant in the *pnp* mutant according to the baySeq algorithm and vice versa (Fig. 3c). This includes every differently expressed gene which fulfills the strict criteria as mentioned above.

The RNA sequencing data was further used to investigate the cellular RNA 3′ elongation. All reads that could not be mapped end-to-end were instead mapped in very sensitive local mode with bowtie2 (version 2.2.6). To increase the quality of the analysis, all reads without a minimal matching sequence of 10 nt at the 5′ end were excluded. Only soft clipped sequences at the 3′ ends of the remaining reads were extracted with awk (version 4.1.3) (Supplementary Fig. S3A, Additional file 1). The overall results are similar for both strains: The lengths of elongated sequences are comparable, the majority of them (95%) is shorter than 39 nt in length. Further, the base frequency for each nucleotide position of the 3′ tail reveals an enrichment of guanine within the first 20 bases (Supplementary Fig. S3B + C + D, Additional file 1). A sequence motif which is related to a PNPase-dependent elongation could not be identified. Since both the lengths and base frequencies of the 3′ tails do not differ in between the analyzed strains, we conclude that the deletion of the KH-S1 domains does not have a major impact on the overall RNA 3′ elongation events in *R. sphaeroides*.

### Levels of regulatory sRNAs are influenced by PNPase

**Fig. 4:**
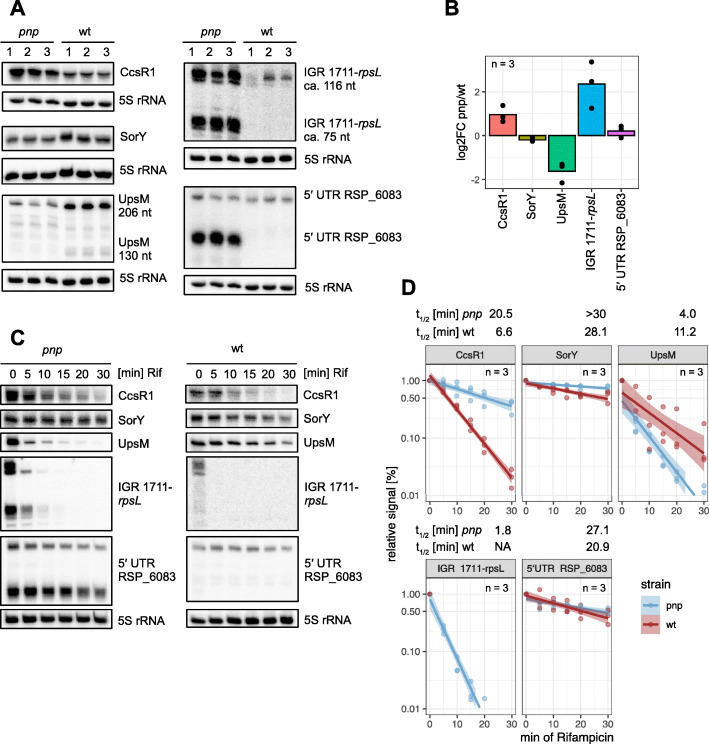
**a + b** Northern blot analysis of the sRNAs CcsR1, SorY, UpsM, IGR_1711_*rpsL* and 5′ UTR RSP_6083. Total RNA was isolated during exponential growth phase (OD_660_ = 0.45) from microaerobic cultures of wild type or *pnp* mutant. Loading control: 5S rRNA. The barchart illustrates the log_2−_fold changes within the indicated sRNAs. *n* = 3. **c** Wild type and *pnp* mutant cells were cultivated under microaerobic conditions until exponential phase. Samples were harvested before (t_0_) and after (t_5_-t_30_) addition of 0.2 mg/ml rifampicin. Total RNA was isolated and used for Northern blot analysis. Loading control: 5S rRNA. **d** The signal intensities for the indicated probes were normalized to the 5S rRNA signal, timepoint t_0_ was set to 1. x-axis: minutes after addition of 0.2 mg/ml rifampicin; y-axis: relative signal intensity. Solid lines represent mean value for biological triplicates, light colors indicate the standard deviation (*n* = 3). Blue: *pnp* mutant; red: wild type. The full-length Northern blots are presented in Supplementary Fig. S4, S5, S6 and S7, Additional file 1

An important effect of the PNPase on levels of small RNAs was reported: the enzyme does not only influence mRNA but also sRNA stability [49–51]. We were especially interested in those sRNAs that are derived from 5′ or 3′ UTRs and wanted to investigate the role of PNPase during the maturation process. For further analysis, we selected five sRNAs which showed a different pattern in the read coverage comparing *pnp* mutant and wild type. Two of them, CcsR1 and SorY, are known to have a regulatory function during the oxidative stress response in *Rhodobacter sphaeroides* [52, 53]. UpsM is processed from the *mraZ* 5′ UTR in a stress-dependent manner by RNase E [54]. The other two sRNAs have not been described so far and their function is still unknown. One is located in the intergenic region between RSP_1711 and *rpsL* and is derived from the *rpsL* 5′ UTR. The second one is derived from the 5′ UTR of RSP_6083. During the exponential growth phase, three of these sRNAs differed in abundance comparing the total RNA from the *pnp* mutant and the wild type strain (Fig. 4a+b). Moreover, processing products of the sRNAs IGR_1711_*rpsL* and 5′ UTR_6083 were prominently enriched in the *pnp* mutant. Interestingly, the abundance of the mature transcript of SorY and 5′ UTR RSP_6083 does not vary between the strains. To further evaluate the sRNA stability, we added rifampicin during the exponential phase and determined the RNA half-lives (Fig. 4c+d). CcsR1, SorY, IGR_1711_*rpsL* and 5′UTR_6083 are strongly stabilized in the mutant lacking PNPase, resulting in prolonged half-lives. In contrast to that, the half-life of UpsM drops form 12.2 min in the wild type to 4.0 min in the *pnp* mutant. The changed stabilities are in agreement with the observed sRNA levels during exponential phase (Fig. 4a). These observations highlight the role of PNPase during the maturation of sRNAs and in the homeostasis of their levels which has been described in *E. coli*. Cameron and De Lay [50] reported a stabilizing function by PNPase on Hfq-dependent sRNAs during the exponential growth phase in *E. coli*. They speculate, that PNPase may for example protect Hfq-bound sRNAs by degrading binding sites for other ribonucleases. Our data show a different trend in *Rhodobacter sphaeroides*, which suggests a mainly destabilizing effect on the selected sRNAs in this study. Even though CcsR1–4, UpsM and SorY are Hfq-dependent [52, 54, 55], CcsR1–4 and SorY are destabilized by PNPase and only UpsM fits the model proposed for *E. coli*. The two UTR-derived sRNAs are also destabilized by PNPase. The reason for these differences remains unidentified. Given this major influence of PNPase on the abundance of regulatory sRNAs, the pleiotropic effect of a *pnp* mutant becomes even more perspicuous.

### Deletion of the KH-S1 domains of PNPase leads mainly to enriched RNA 3′ ends

As a 3′-to-5′ exoribunuclease, PNPase plays an important role in RNA turnover and decay from the 3′ end. Therefore, we analyzed how the RNA 3′ ends differ in abundance comparing the *pnp* mutant to the wild type. For this study we developed XPEAP, an analysis pipeline. It allows the detection of RNA 5′ or 3′ ends in prokaryotic NGS data and covers all relevant steps form data preprocessing to the final statistical analysis. As input the raw read files, three replicates of each strain, were used. After trimming and read alignment to the reference genome, READemption’s subcommand *coverage* was used to generate coverage files which contain the nucleotide positions of the 3′ end bases of each aligned read. The coverage files from the plus and minus strand were integrated in one data set. Subsequently all nucleotide positions that did not exhibit a coverage of ≥10 in at least one of the analyzed libraries were excluded. We further calculated the ratio of the 3′ end coverage and the full read coverage for every replicate and every position. To improve the signal noise ratio, only positions with a ratio higher than 0.05 were kept. A DESeq2 analysis was conducted based on this nucleotide-wise coverage files to detect differences within the two strains. All positions which showed a log_2_-fold change ≤ − 1 or ≥ + 1 and an adjusted *p*-value ≤0.05 (Benjamini Hochberg algorithm) were kept for further analysis, the other positions were rejected. Due to the fact that the 3′-to-5′ end processing is a dynamic process, it is supposed that in some cases several 3′ ends per RNA molecule will be detected. This is why we decided to merge all nucleotide positions within a range of 3 nt with BEDtools’ subcommand *merge*. The range of mapped positions which belong to one 3′ end is defined as distribution size. All resulting positions are regarded as true differential 3′ ends.

**Fig. 5:**
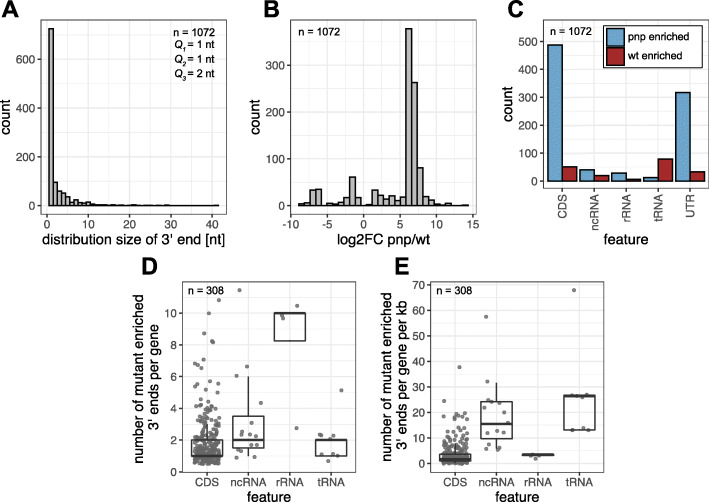
The PNPase mutation leads to a change in RNA 3′ end composition. **a** Histogram illustrating the different distribution sizes of all 1072 differential 3′ ends. *Q*_1_ = 1 nt; *Q*_2_ = 1 nt; *Q*_3_ = 3 nt; arithmetic mean = 3.86 nt. **b** Distribution of the log_2_-fold changes of all differential 3′ ends, comparing the *pnp* mutant strain and the wild type. 82% of the 3′ ends are enriched in the mutant. **c** All 3′ ends were assigned to genes or UTRs according to their genomic position and then grouped in feature classes. Most 3′ ends in CDS (coding sequences) and UTRs were enriched in the *pnp* mutant, but more tRNA 3′ ends were detected in the wild type. **d + e** The boxplots show the absolute number of 3′ ends enriched in the mutant per gene **d** and the relative number (normalized to the gene length) (**e**). Overall 306 genes were observed to harbour at least one 3′ end enriched in the mutant. Every dot represents one gene

In total 1072 differential 3′ ends could be detected, the majority of them (around 68%) were mapped to a single position (Fig. 5a). By far most of all 3′ ends (82%) were strongly enriched in the *pnp* mutant strain showing a log_2_-fold change between + 5 and + 9 (Fig. 5b). These ends represent either termination sites or arise from cleavage by endoribonucleases and are further processed by PNPase in the wild type. The ends were strand-specifically assigned to the different feature classes according to their genomic position with BEDtools *intersect*. All 3′ ends that did not overlap with any feature were classified in the group UTR. This group may also contain additional sRNAs that have not yet been identified. The most prominent changes could be observed within the coding sequences and the untranslated regions: In both feature classes, around 10 times more RNA 3′ ends were enriched in the *pnp* mutant compared to the wild type (Fig. 5c). These 885 ends are interpreted as PNPase-degraded. To search for putative motifs, the 15 nt upstream sequence of every *pnp* enriched differential 3′ end was extracted with BEDtools *getfasta*. Only non-overlapping 3′ ends were kept for the following analysis to reduce bias during the motif analysis. No binding motif or consensus sequence could be found with MEME Suite (version 5.3.0).

We further detected 189 3′ ends which are enriched in the wild type. One possible explanation for this observation is, that these RNAs are degraded by PNPase from the 3′ end, but at the detected positions stable secondary structures prevent further RNA 3′-to-5′ end degradation in the wild type. For a test of this hypothesis all sequences within windows of 20 nt, 30 nt, 40 nt and 50 nt upstream of the detected wild type enriched 3′ ends were extracted as described above. As a control we selected sequences of the same length but located downstream of the 3′ ends (Supplementary Fig. S8A, Additional file 1). These RNA sequences are supposed to have no effect on the stop of PNPase decay since they are properly degraded. RNAfold (version 2.4.17) was used to compute the minimal folding energy (MFE) of every sequence. Independently of the window size, the distributions of both groups are highly similar and no shift to low MFE values was observable in the upstream sequences (Supplementary Fig. S8B, Additional file 1). But remarkably, the number of unstructured sequences (MFE = 0.0 kcal/mol) was three times higher downstream of the 3′ ends (*n* = 29, 13.8%) compared to the regions upstream of the 3′ ends (*n* = 9, 4.8%) (Supplementary Fig. S8C, Additional file 1). We therefore conclude that unstructured sequences may at least enhance degradation by PNPase while highly structured RNA sequences may facilitate a stop of decay. Moreover, other still unknown factors, e.g. binding proteins, are likely to influence degradation by PNPase. Using MEME, no recurring motif that could hint to conserved binding sites was detectable in the upstream sequences.

The group of tRNAs exhibited less differential 3′ ends in the *pnp* mutant strain, all with rather minor log_2_-fold changes (median − 1.3). This affects 32 of in total 54 tRNAs. We observed characteristic 3′ ends which show a clear edge in the wild type RNA coverage profile of the tRNA^Gly^ and tRNA^Leu^ (see Supplementary Fig. S9A, Additional file 1). The other tRNAs coverage profiles of the wild type harbour only minor edges but are stronger sloped than in the *pnp* mutant (see Supplementary Fig. S9B, Additional file 1). The reason for these differences still remains concealed. Nevertheless, the detection of those 3′ ends depicts the high sensitivity of XPEAP. The influence of the *E. coli* PNPase on tRNA maturation and degradation has been investigated intensively. PNPase is involved in the repair process of several tRNAs [56], although this enzyme does not affect the tRNA poly(A) tail length [57]. Furthermore, both PNPase and RNase II remove the Rho-independent terminator structure of the leuX tRNA [58]. In chloroplasts of *A. thaliana*, the PNPase activity is directly linked to the decay of tRNAs [59].

Moreover, our data suggest that PNPase-dependent degradation is not limited to only one site per gene. In many cases, more than one RNA 3′ end per gene was enriched in the mutant (Fig. 5d+e). This affects in particular the 23S rRNAs, but also several mRNAs and ncRNAs. This is not surprising, since often several RNase E cleavage sites per gene could be detected in *Rhodobacter sphaeroides*, but also in *Salmonella enterica* [6, 7]. After RNase E cleavage, the RNA fragments become potential new substrates for PNPase.

It should be mentioned that some of the 1072 differential 3′ ends may arise from a stop of the sequencing reaction after about 75 nucleotides in the single-end sequencing. 3′ ends at such a distance from the 5′ end account for only 4.8% of all differential 3′ ends. Moreover, this number also contains real 3′ ends as depicted in Fig. 4: as predicted, a 75 nt RNA derived from IGR 1711-*rpsL* occurs only in the mutant but not in the wild type.

### Intersection analysis

Multiple ribonucleases are involved in RNA processing and turnover. In many organisms, the ribosomal RNA maturation requires an initial endonucleolytic cleavage of the long nascent precursor transcript by RNase III. This is followed by further enzymatic reactions, performed for example by RNase E, J, G and various other enzymes. PNPase can process those RNA species which are newly generated by endonucleases from the 3′ end. Furthermore, both PNPase and RNase E are part of the *E. coli* degradosome and work together in RNA degradation. In the *R. capsulatus* degradosom fraction PNPase activity could be detected, but only a small amount [11]. To get more insight into the interplay of RNase E, RNase III and PNPase we analyzed the correlation of 3′ ends from the wild type, the *pnp* mutant, an RNase III mutant and a strain with reduced RNase E activity. In the RNase III mutant strain, the *rnc* gene was removed by homologous recombination [24]. Since RNase E is an essential enzyme in *Rhodobacter sphaeroides*, the *rne* mutant strain was achieved by replacing the native *rne* by the gene of the thermosensitive RNase E from *E. coli* [54]. At 32 °C the enzymatic activity is already reduced and is even more reduced at 42 °C [7].

**Fig. 6:**
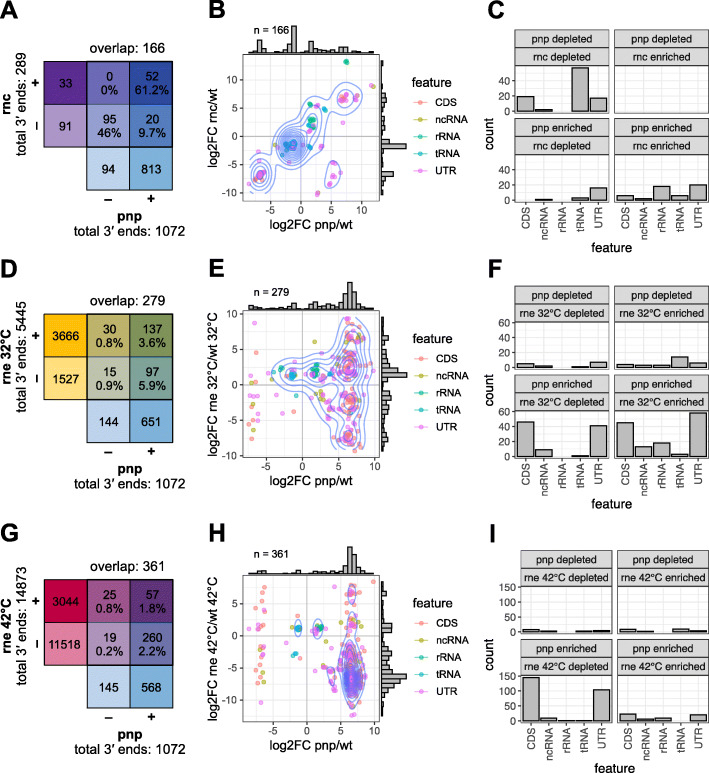
Many differential 3′ ends found in the PNPase mutant can also be detected in the RNase III or RNase E mutant strain at the same genomic position. **a, d, g** Venn diagrams illustrating the total number of differential 3′ ends found in each mutant strain in comparison to the wild type (+: mutant enriched; −: mutant depleted) and the number differential 3′ ends which could be mapped to the same genomic position. Percentage: number of 3′ ends in respective quadrant devided by the total number of *rnc*/*rne* 32 °C/*rne* 42 °C enriched or depleted 3′ ends. Blue: *pnp* mutant; violett: *rnc* mutant; yellow: *rne* mutant at 32 °C; red: *rne* mutant at 42 °C. **b, e, h** Scatterplots depicting the intersecting analysis, every dot represents one differential 3′ end. x-axis: log_2_-fold change *pnp* mutant versus wild type; y-axis: log_2_-fold change *rnc/rne* mutant versus wild type. Feature classes harboring the observed 3′ ends are color coded. Orange: CDS; yellow: ncRNA; green: rRNA; blue: tRNA; violett: UTR. Blue lines represent a 2D density function. The histograms (grey) illustrate the distribution of intersecting 3′ end log_2_-fold changes of the compared strains. **d, f, i** Histograms display the number of intersecting 3′ ends, which are grouped in mutant enriched/depleted and feature classes. x-axis: feature class; y-axis: count

First, the differential 3′ ends were detected as described in the previous section (see Additional file 3 for the full list of detected RNA 3′ ends). For the comparison of a mutant to the corresponding wild type strain, we only analyzed data that was obtained from the very same sequencing chip. Next, the overlap between the detected 3′ ends was investigated with BEDtools’ subcommand *window*. Using this function, a window of 1 nt upstream and 1 nt downstream of every differential *pnp* mutant 3′ end was set prior to the intersection analysis to compensate a potential inaccuracy during 3′ end determination. Two hundred eighty-nine differential 3′ ends could be detected in the *rnc* mutant, more than half of them (166; 57%) were uniquely overlapping with PNPase dependent 3′ ends (Fig. 6a). Within this group, no 3′ end showed a reduced abundance in the *pnp* mutant and an increased abundance in the *rnc* mutant strain (Fig. 6b). By far the highest number of overlapping ends could be found in tRNAs (Fig. 6c). Taken together, 9.7% of all RNase III generated RNA 3′ ends are further processed by PNPase (Fig. 6a + b: quadrant *rnc* depleted, *pnp* enriched). Although in *E. coli* RNase III is involved in *pnp* mRNA processing [20, 60], we could not observe any effect on the *pnp* mRNA levels in the RNase III mutant strain in *Rhodobacter sphaeroides*.

A much greater number of total 3′ ends was identified to be RNase E-dependent: 5445 at 32 °C and 14,873 at 42 °C (Fig. 6d+g). At the permissive temperature, the majority of differential 3′ ends could be assigned to CDS and UTR (Fig. 6e+f). At the non-permissive temperature more than 2.5% of all differential *pnp* mutant RNA 3′ ends (279 in total, 271 unique *pnp* mutant ends) overlap with those differential 3′ ends identified in the *rne* mutant. Plotting the log_2_-fold changes reveals a specific pattern: 75% of all intersecting 3′ ends are *pnp* enriched and *rne* depleted (Fig. 6h), suggesting that these 3′ ends are generated by RNase E cleavage and further removed by PNPase. This group is mainly composed of ends located in CDS and UTRs, but also in several ncRNAs (Fig. 6i). Interestingly only 5.9% of all 3′ ends which are generated by RNase E are further degraded by PNPase. Such a low fraction of around 6% was also observed in *S. pyogenes* [31]. We hypothesize, that a portion of the RNase E generated 3′ ends can at least partly be trimmed by other 3′-to-5′ exonucleases and thus are not detected in the *pnp* mutant strain. Also, stable secondary structures at the newly generated 3′ ends may prevent PNPase degradation. At the permissive temperature, the *pnp* mRNA levels do not differ, whereas at 42 °C the *pnp* mRNA levels are more than doubled (log_2_-fold change = 1.75, comparison *rne* mutant versus wild type) strongly supporting an effect of RNase E on *pnp* stability.

We further elucidated the statistical significance of the observed overlapping 3′ ends. For every comparison of detected 3′ ends (*pnp* mutant versus *rnc*/*rne* mutant) BEDtools’ subcommand *fisher* was used to first compute the number of possible 3′ ends, taking into account the genome size and the individual distribution sizes of determined 3′ ends. Second, the number of overlaps and non-overlaps was computed and the Fisher’s exact test applied. For a possible number of 633,092 (*pnp* versus *rnc*), 748,506 (*pnp* versus *rne* 32 °C) and 667,153 (*pnp* versus *rne* 42 °C) RNA 3′ ends the resulting two-tail *p*-value are close or equal to 0 in each case (0, 7.3 × 10^− 241^, 2.9 × 10^− 239^). This strongly suggests a higher number of RNA 3′ ends at the same genomic positions than would be expected, if the given 3′ ends would be randomly distributed within the genome.

The total number of detected 3′ ends as well as the overlapping 3′ ends differ within the strains analyzed in this study. It cannot be excluded, that this observation may be influenced by a different number of uniquely aligned reads: The samples of the RNase E mutant and the corresponding wild type strain previously published [7] showed on average a four times higher number of uniquely aligned reads than the samples sequenced for this study (PNPase mutant, RNase III mutant and wild type strain). On the other hand, although Lécrivain et al. [61] used a somewhat different algorithm and parameters to detect differential 3′ ends in *Streptococcus pyogenes*, the number of identified 3′ ends (wild type enriched: 183; *pnp* mutant enriched: 1255) is strikingly similar to our findings in the Gram-negative organism *R. sphaeroides* (wild type enriched: 189; *pnp* mutant enriched: 885). This similarity strongly supports the reliability of the data obtained in this study and suggests valid differences in between the analyzed strains.

**Fig. 7:**
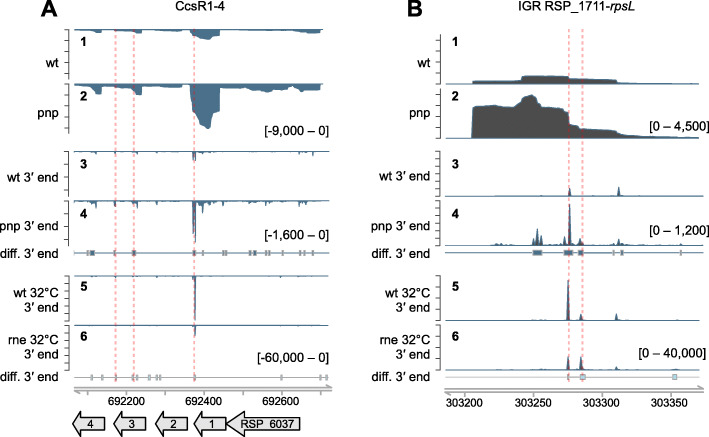
The sRNA levels of CcsR1–4 and IGR_RSP_1711_*rpsL* are modulated by RNase E cleavage and PNPase processing. XPEAP reveals overlapping differential 3′ ends detected in the RNAs CcsR1–4 (**a**) and IGR_RSP_1711_*rpsL* (**b**). Panel 1 and 2: RNA-Seq full coverage (wild type and *pnp* mutant strain). Panel 3 and 4: 3′ end coverage (wild type and *pnp* mutant strain). Panel 5 and 6: 3′ end coverage (wild type and *rne* mutant strain at 32 °C). Small boxes indicate positions of true differential 3′ ends computed with XPEAP. Red lines: overlapping differential 3′ ends. Y-axis count scales are indicated in square brackets

To illustrate these overlapping 3′ ends we chose two of the previously shown sRNAs. Several differential 3′ ends can be found in the sRNAs CcsR1–4, which are derived from one co-transcript by RNase E cleavage [52]. The read coverage depicts a strong enrichment of CcsR1–4 in the *pnp* mutant (Fig. 7a), which agrees with the Northern Blot data (Fig. 4a). Several *pnp* mutant enriched 3′ ends have been detected. Three of them overlap with those RNA 3′ ends which are depleted in the *rne* mutant in comparison to the wild type, even at 32 °C (Fig. 7a, highlighted in red color). The read coverage of the sRNA IGR_RSP_1711_*rpsL* reveals, that the first 75 bases of the RNA are enriched in the *pnp* mutant (Fig. 7b). This shorter fragment was also detected by Northern blot analysis (Fig. 4a). Comparing wild type and the RNase E deficient strain, a 3′ end depleted in the mutant could be found at the very same site. Despite some changes in overall abundance, no RNase III-dependent differential 3′ ends were detectable. For both of the two described sRNAs, a similar processing pattern is proposed. The initial transcript is first processed by RNase E, indicated by the depleted 3′ ends in the *rne* mutant. Followed by this cleavage, the remaining RNA molecule is further degraded by PNPase thus leading to enriched 3′ ends in the *pnp* mutant. This observation is in agreement with previous studies in Gram-negative (reviewed in [29, 30]) as well as in Gram-positive bacteria. In *Streptococcus pyogenes* for example, the endonuclease RNase Y generates new RNA 3′ ends, which are subsequently trimmed by PNPase [31]. PNPase seems not to be a component of the degradosome of the related species *R. capsulatus* [11], which most likely also accounts for *R. sphaeroides*. Even though this direct interaction between RNase E and PNPase may be lacking, our data provide strong evidence that stepwise RNA processing is mediated by these enzymes also in *Rhodobacter.*

## Conclusion

In this study we characterized the function of PNPase in the Gram-negative alpha-proteobacterium *Rhodobacter sphaeroides* and shed light on the interplay of RNase E, RNase III and PNPase during RNA degradation. A lack of the KH-S1 RNA binding domain leads to severe effects on growth behavior, temperature stress tolerance, pigmentation and the bacteriochlorophyll *a* content. Total RNA sequencing illustrates a high impact of PNPase on levels of diverse RNAs, in particular on tRNAs and rRNAs. We could further demonstrate, that the stability of several regulatory sRNAs relies on PNPase function, thus leading to altered levels during exponential growth phase. Next, the global comparison of differential RNA 3′ ends identified in the *pnp* and *rnc* mutant strain as well as in a strain with reduced RNase E activity is in agreement with a sequential processing of transcripts: 5.9% of all RNase E and 9.7% of all RNase III generated RNA 3′ ends are afterwards trimmed by PNPase.

## Supplementary Information

**Additional file 1: Table S1.** Strains used in this study. **Table S2.** Overview of all oligonucleotide probes and corresponding sequences that were used in this study. **Figure S1.** Scatterplot of the principal component analysis, which was performed using DESeq2. Each of the colored dots (*pnp* mutant strain: red; wild type: blue) represents one replicate. **Figure S2.** Comparing the *pnp* mutant to the wildtype, the RNAs with decreased (A) or increased abundance (B) could not be assigned to specific orthologous groups of encoded proteins (COG). x-axis: relative number of observations per group [%]; y-axis: COG category. **Figure S3.** The 3′ elongated RNA sequences are similar in the wild type and the *pnp* mutant strain. A) Schematic overview of the sequence extraction procedure. The lengths of all non end-to-end mapped reads (B) and of all tail sequences (C) do not differ. In both strains the first part of the tail (around 20 nt in length) is guanine rich (D). **Figure S4.** Full size Northern blots of the depicted images in Fig. 4. with probes CcsR1 (A), SorY (B), UpsM (C), IGR_1711_*rpsL* (D) and 5′ UTR RSP_6083 (E). Red frames mark the selected sections. **Figure S5.** Full size Northern blots of the depicted half-life images in Fig. 4. with probes CcsR1 (A) and SorY (B). Red frames mark the selected sections. **Figure S6.** Full size Northern blots of the depicted half-life images in Fig. 4, with probes UpsM (A) and IGR_1711_*rpsL* (B). Red frames mark the selected sections. **Figure S7.** Full size Northern blots of the depicted half-life images in Fig. 4 with probe 5′ UTR RSP_6083. Red frames mark the selected sections. **Figure S8.** A) Sequence windows of 20, 30, 40 or 50 nt upstream and downstream of every wild type enriched 3′ end were extracted. B) Every dot in the boxplots depicts the minimal folding energy (MFE) [kcal/mol] of one RNA sequence computed with RNAfold. C) The number of unstructured sequences (MFE = 0.0 kcal/mol) is increased in the group of 20 nt downstream control sequences. **Figure S9.** Representative RNA full coverage and 3′ end coverage profiles of two selected tRNAs in the wild type and the *pnp* mutant strain. The full coverage of tRNAs can exhibit characteristic 3′ ends as shown for tRNA^Leu^ (A) or 3′ ends which are detected because of minor edges and a different slope (tRNA^Pro^, B).

**Additional file 2: **Supplementary tables of differential gene expression. **Table S3.** DESeq2 analysis results comparing the differential gene expression in the *pnp* mutant strain and the wild type. **Table S4.** BaySeq analysis results comparing the differential gene expression in the *pnp* mutant strain and the wild type.

**Additional file 3: **Supplementary tables of all differential RNA 3′ ends. **Table S4.** Differential RNA 3′ ends comparing the *pnp* mutant strain and the wild type. **Table S5.** Differential RNA 3′ ends comparing the *rnc* mutant strain and the wild type. **Table S6.** Differential RNA 3′ ends comparing the *rne* mutant strain (32 °C) and the wild type (32 °C). **Table S7.** Differential RNA 3′ ends comparing the *rne* mutant strain (42 °C) and the wild type (42 °C).

## Data Availability

The datasets generated and analyzed during the current study are available in the NCBI Gene Expression Omnibus repository (GSE156818 and GSE71844).
