# Supplementary material for: Impact of PNPase on the transcriptome of Rhodobacter sphaeroides and its cooperation with RNase III and RNase E
Source: BMC Genomics. 2021 Feb 6;22:106. doi: 10.1186/s12864-021-07409-4 (PMC7866481; doi:10.1186/s12864-021-07409-4)
Supplement: Supplementary file 1 — Additional file 1: Table S1. Strains used in this study. Table S2. Overview of all oligonucleotide probes and corresponding sequences that were used in this study. Figure S1. Scatterplot of the principal component analysis, which was performed using DESeq2. Each of the colored dots (pnp mutant strain: red; wild type: blue) represents one replicate. Figure S2. Comparing the pnp mutant to the wildtype, the RNAs with decreased (A) or increased abundance (B) could not be assigned to specific orthologous groups of encoded proteins (COG). x-axis: relative number of observations per group [%]; y-axis: COG category. Figure S3. The 3′ elongated RNA sequences are similar in the wild type and the pnp mutant strain. A) Schematic overview of the sequence extraction procedure. The lengths of all non end-to-end mapped reads (B) and of all tail sequences (C) do not differ. In both strains the first part of the tail (around 20 nt in length) is guanine rich (D). Figure S4. Full size Northern blots of the depicted images in Fig. 4. with probes CcsR1 (A), SorY (B), UpsM (C), IGR_1711_rpsL (D) and 5′ UTR RSP_6083 (E). Red frames mark the selected sections. Figure S5. Full size Northern blots of the depicted half-life images in Fig. 4. with probes CcsR1 (A) and SorY (B). Red frames mark the selected sections. Figure S6. Full size Northern blots of the depicted half-life images in Fig. 4, with probes UpsM (A) and IGR_1711_rpsL (B). Red frames mark the selected sections. Figure S7. Full size Northern blots of the depicted half-life images in Fig. 4 with probe 5′ UTR RSP_6083. Red frames mark the selected sections. Figure S8. A) Sequence windows of 20, 30, 40 or 50 nt upstream and downstream of every wild type enriched 3′ end were extracted. B) Every dot in the boxplots depicts the minimal folding energy (MFE) [kcal/mol] of one RNA sequence computed with RNAfold. C) The number of unstructured sequences (MFE = 0.0 kcal/mol) is increased in the group of 20 nt downstream control sequences. Fig [file 12864_2021_7409_MOESM1_ESM.pdf]

Table S1

Rhodobacter sphaeroides strain	description	reference
2.4.1	wild type	[61]
rne^{ts}	Δ(rne::rne$^{E.c.ts}$;Sp)	[53]
Δrnc	Δ(rnc::Km)	[24]
pnpΔKH-S1	Δ(pnp_KH-S1::Gm)	this study

Table S2

name	sequence 5'-3'	reference
p_CcsR1	CGTCGCCGCTGCTGCTACAGGTC	[51]
p-0682 (UpsM)	GACTCAGGTGGTCGCCAGATACC	[53]
p-1543	ATGAAGCGGACGAGAGAACCCTC	[52]
probe_RSP1711_rpsL	CGGATCATGACCACTACAGTTAACT	this study
probe_RSP6083_5UTR	GGAAAGGCTACGTTCGAAAGTTAGG	this study

Fig S1

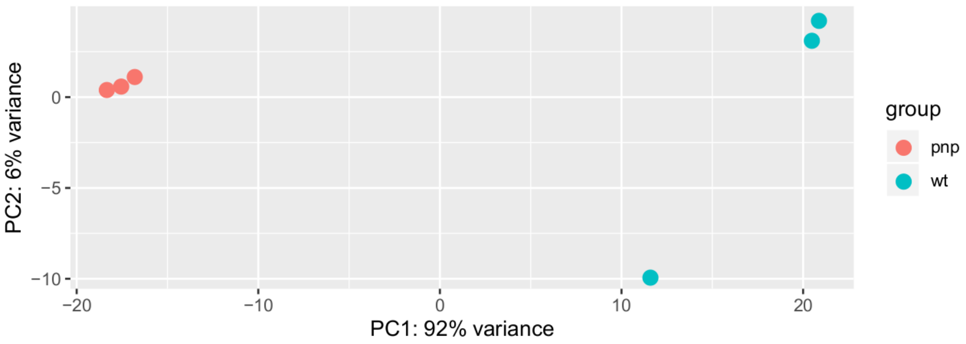

Fig S2

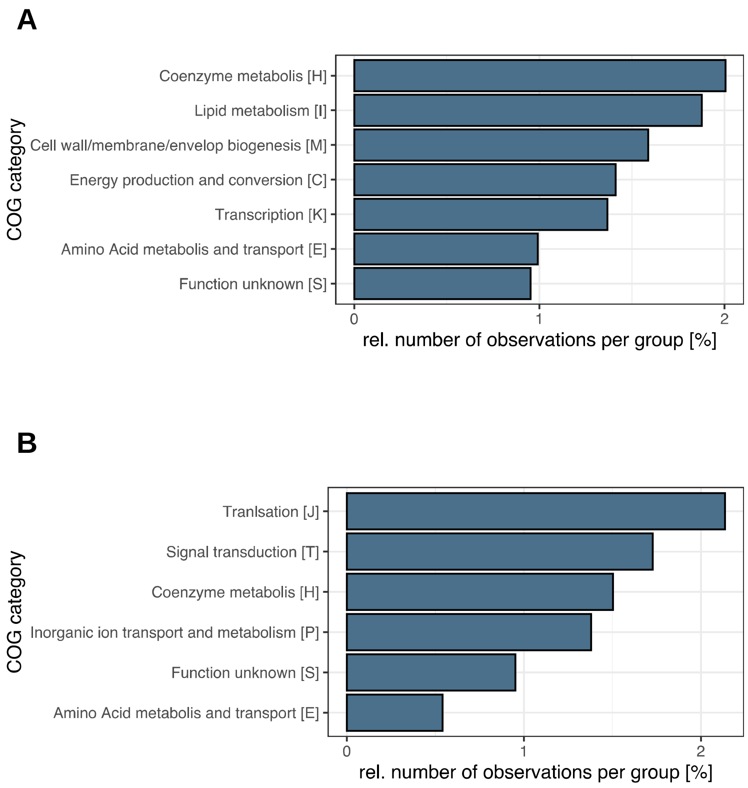
A
COG category
Coenzyme metabolis [H]
Lipid metabolism [I]
Cell wall/membrane/envelop biogenesis [M]
Energy production and conversion [C]
Transcription [K]
Amino Acid metabolis and transport [E]
Function unknown [S]
0
1
2
rel. number of observations per group [%]
B
COG category
Tranlsation [J]
Signal transduction [T]
Coenzyme metabolis [H]
Inorganic ion transport and metabolism [P]
Function unknown [S]
Amino Acid metabolis and transport [E]
0
1
2
rel. number of observations per group [%]

Fig S3

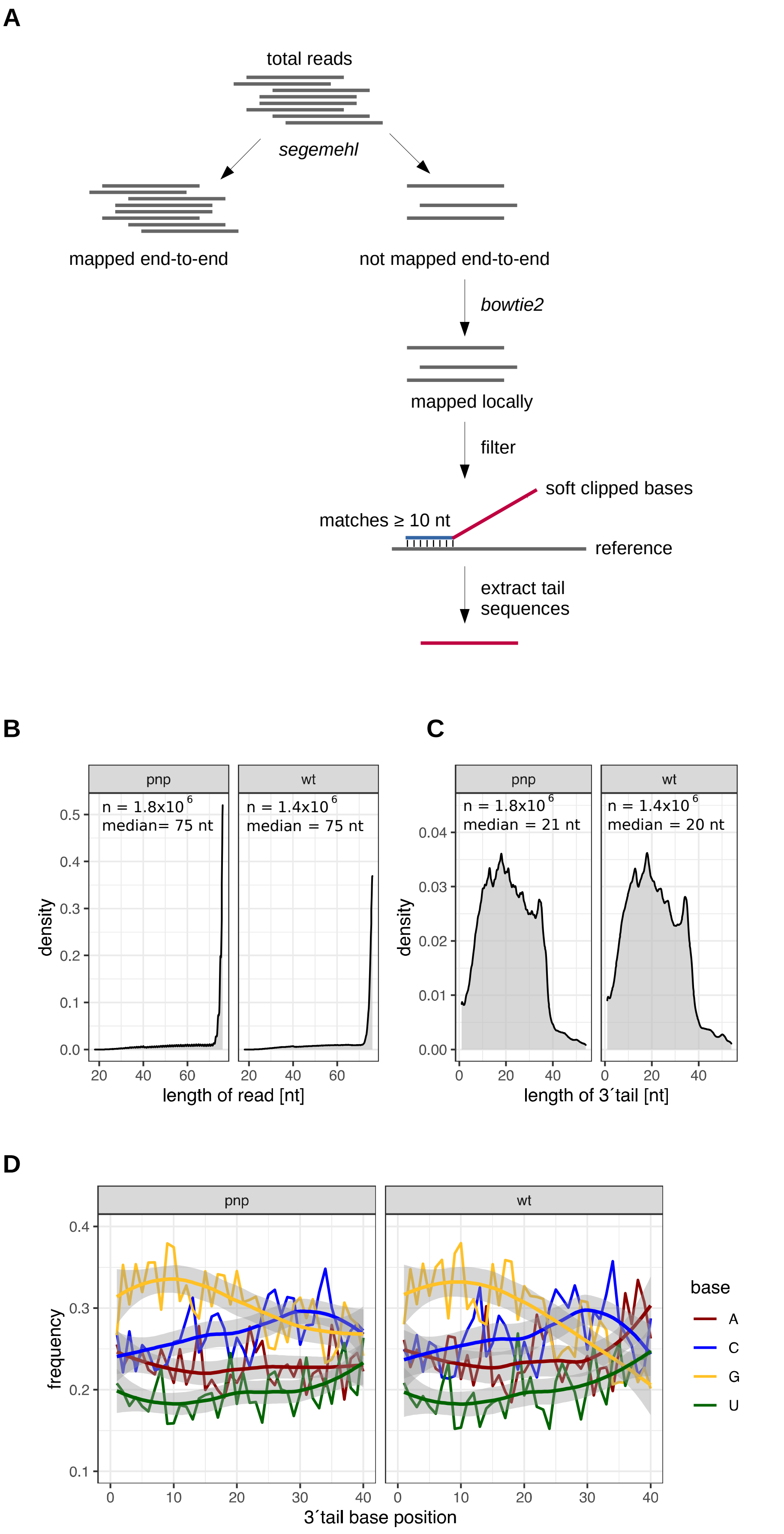

A
total reads
segemehl
mapped end-to-end
not mapped end-to-end
bowtie2
mapped locally
filter
soft clipped bases
matches ≥ 10 nt
reference
extract tail
sequences
B
pnp
wt
n = 1.8x10 6
median= 75 nt
n = 1.4x10 6
median = 75 nt
density
length of read [nt]
C
pnp
wt
n = 1.8x10 6
median = 21 nt
n = 1.4x10 6
median = 20 nt
density
length of 3´tail [nt]
D
pnp
wt
frequency
3´tail base position
base
A
C
G
U

Fig S4

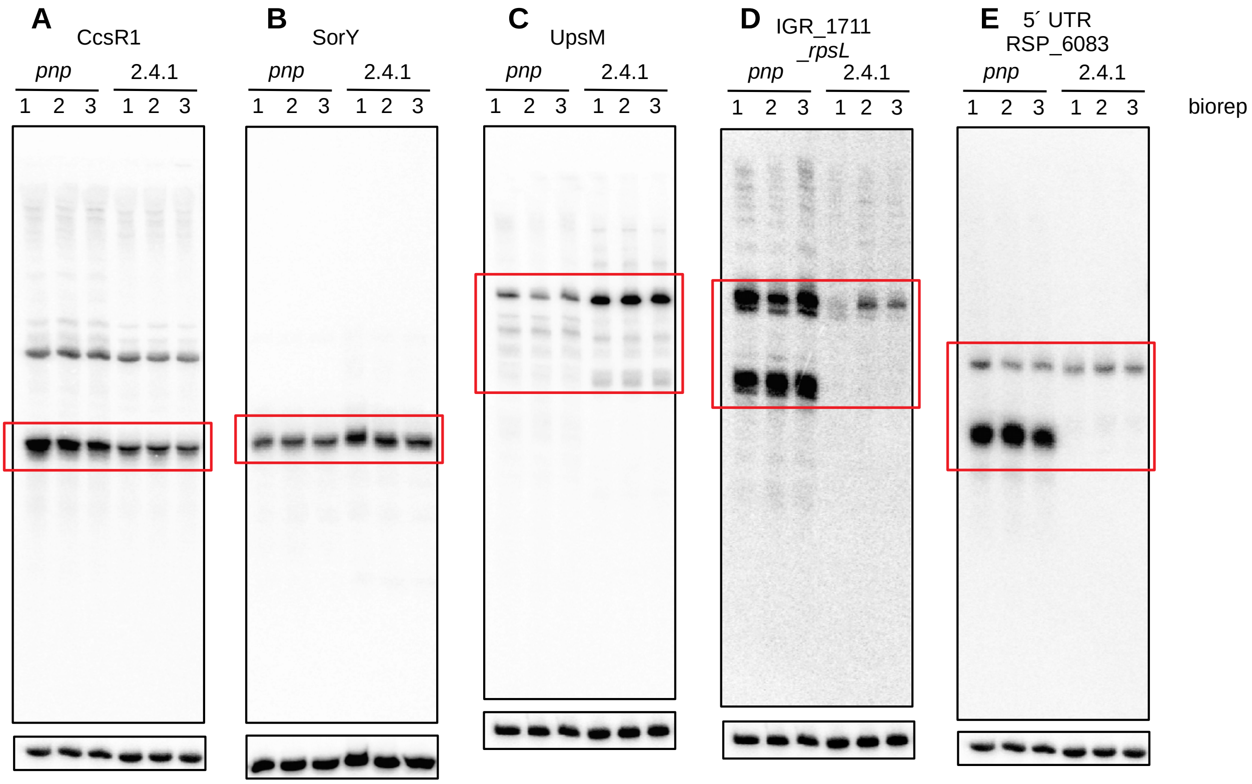
A
CcsR1
B
SorY
C
UpsM
D
IGR_1711
_rpsL
E
5´ UTR
RSP_6083
pnp
2.4.1
1 2 3 1 2 3
biorep

Fig S5

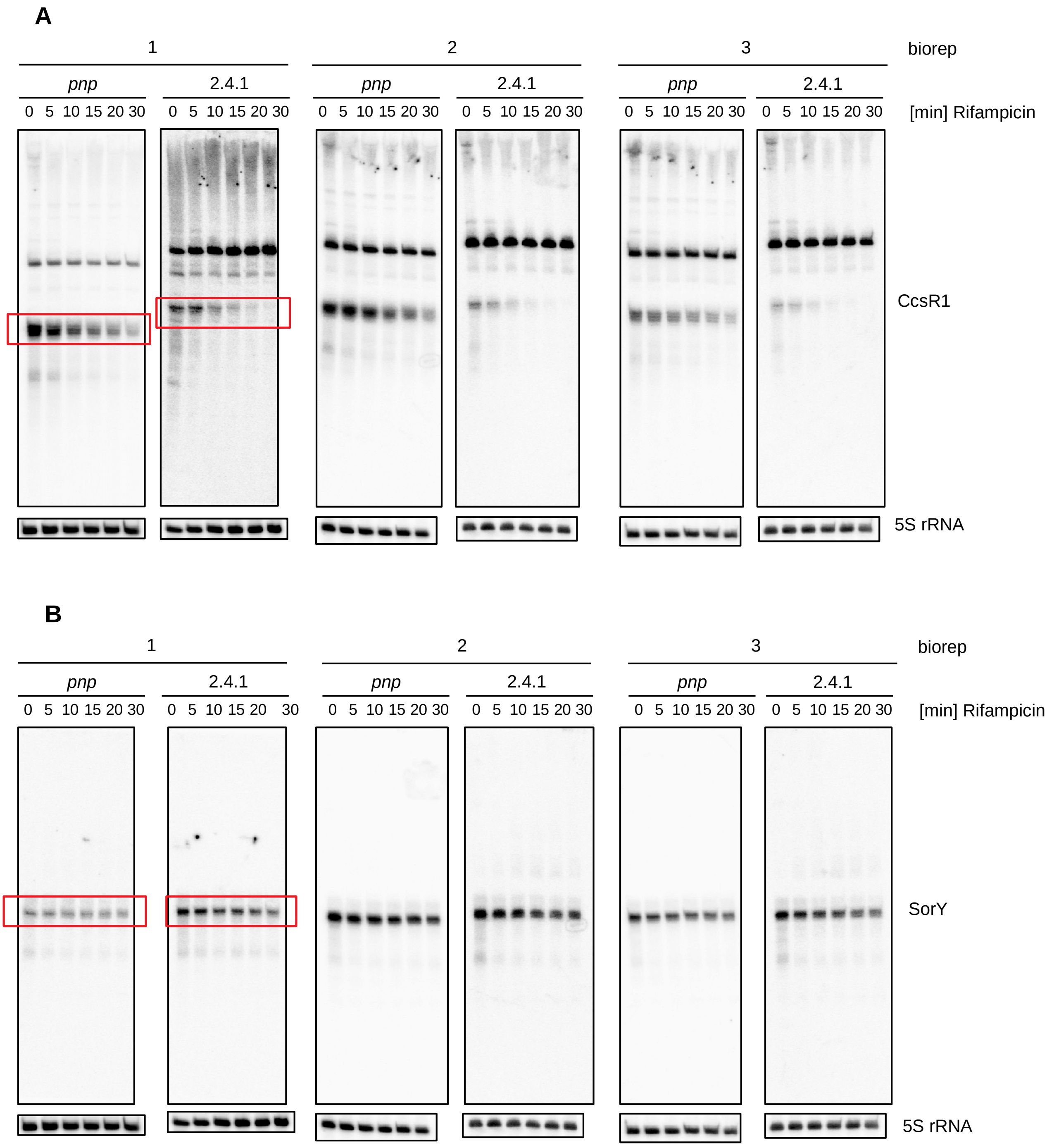
A
1
2
3
biorep
pnp
2.4.1
0 5 10 15 20 30
[min] Rifampicin
CcsR1
5S rRNA
B
SorY

Fig S6

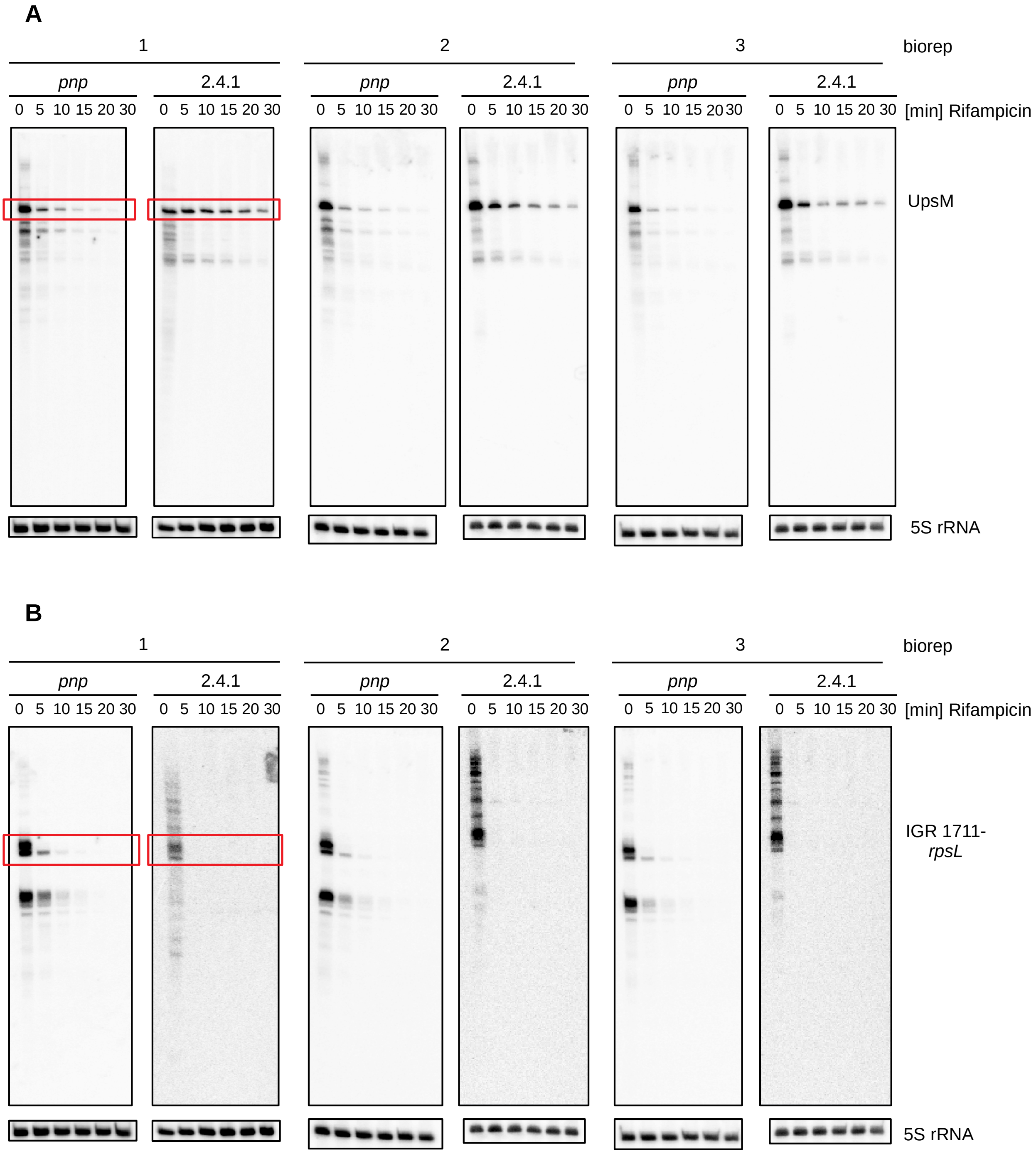
A
1
2
3
biorep
pnp
2.4.1
0 5 10 15 20 30
[min] Rifampicin
UpsM
5S rRNA
B
1
2
3
biorep
pnp
2.4.1
0 5 10 15 20 30
[min] Rifampicin
IGR 1711-
rpsL
5S rRNA

Fig S7

A

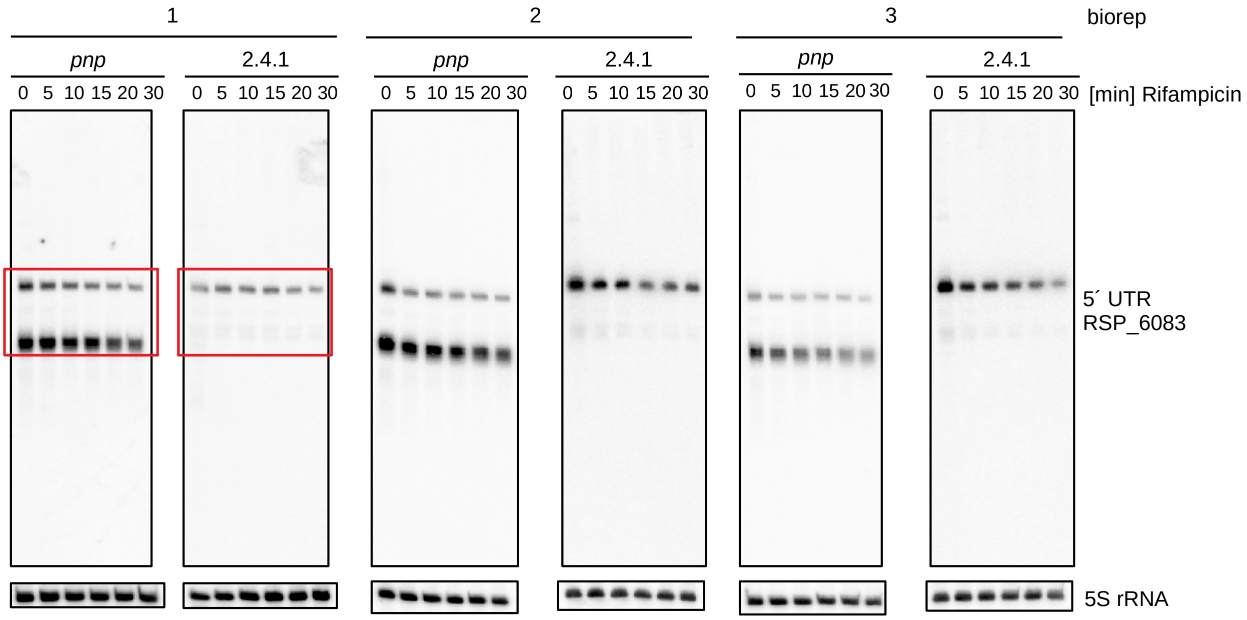
1
2
3
biorep
pnp
2.4.1
pnp
2.4.1
pnp
2.4.1
0 5 10 15 20 30
0 5 10 15 20 30
0 5 10 15 20 30
0 5 10 15 20 30
0 5 10 15 20 30
0 5 10 15 20 30
[min] Rifampicin
5´ UTR
RSP_6083
5S rRNA

Fig S8

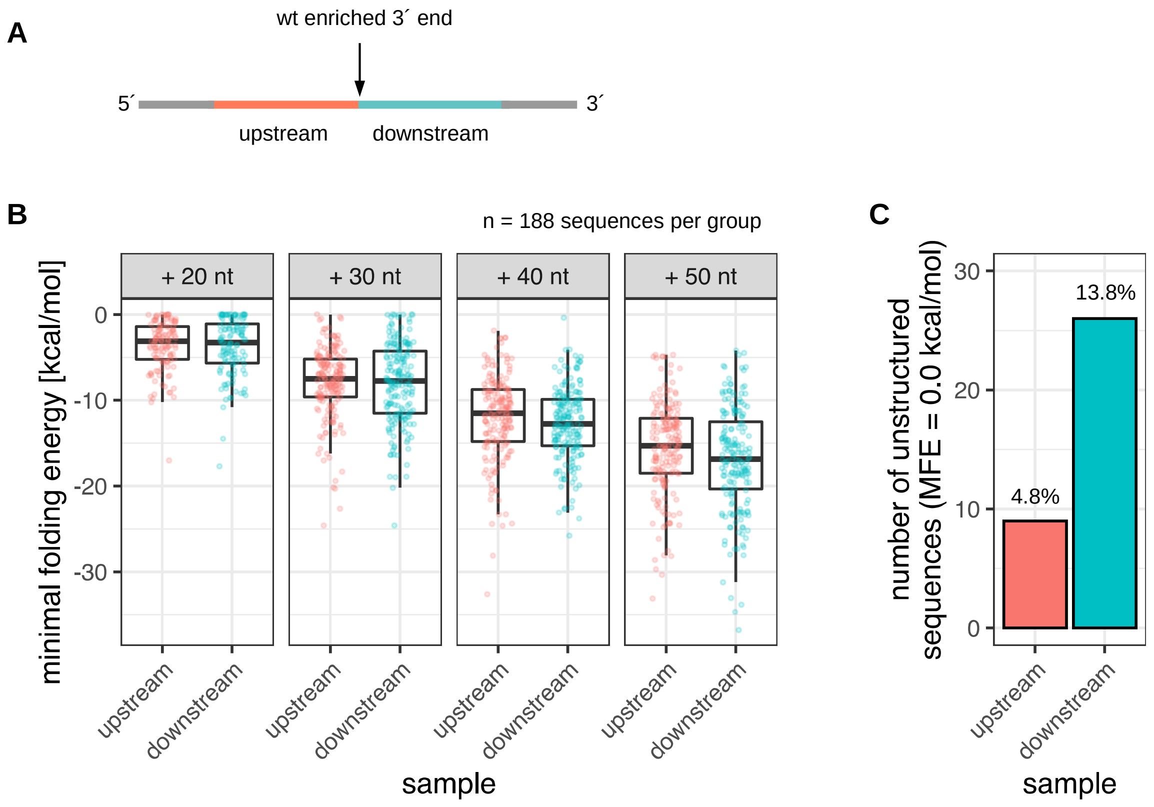
A
wt enriched 3´ end
5´
3´
upstream
downstream
B
n = 188 sequences per group
+ 20 nt
+ 30 nt
+ 40 nt
+ 50 nt
minimal folding energy [kcal/mol]
0
-10
-20
-30
upstream
downstream
sample
C
number of unstructured sequences (MFE = 0.0 kcal/mol)
30
20
10
0
4.8%
13.8%
upstream
downstream
sample

Fig S9

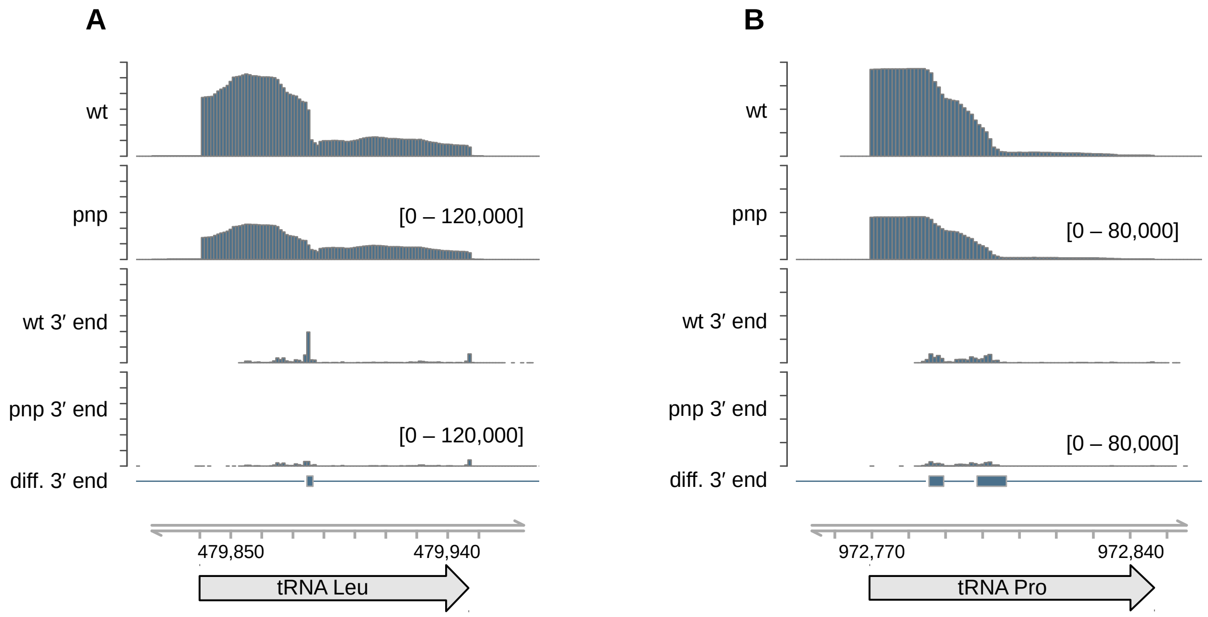
A
wt
pnp
[0 – 120,000]
wt 3′ end
pnp 3′ end
[0 – 120,000]
diff. 3′ end
479,850
479,940
tRNA Leu
B
wt
pnp
[0 – 80,000]
wt 3′ end
pnp 3′ end
[0 – 80,000]
diff. 3′ end
972,770
972,840
tRNA Pro
